# Lipophilic vs. hydrophilic statins and psychiatric hospitalizations and emergency room visits in US Veterans with schizophrenia and bipolar disorder

**DOI:** 10.1515/pteridines-2020-0028

**Published:** 2021-09-23

**Authors:** Teodor T. Postolache, Deborah R. Medoff, Clayton H. Brown, Li Juan Fang, Sanjaya K. Upadhyaya, Christopher A. Lowry, Michael Miller, Julie A. Kreyenbuhl

**Affiliations:** VISN 5 Capitol Health Care Network Mental Illness Research Education and Clinical Center (MIRECC), U.S. Department of Veterans Affairs, Baltimore, MD 21201, United States of America; Rocky Mountain Mental Illness Research Education and Clinical Center (MIRECC), U.S. Department of Veterans Affairs, Aurora, CO 80045, United States of America; Department of Psychiatry, University of Maryland School of Medicine, Baltimore, MD 21201, United States of America; Military and Veteran Microbiome: Consortium for Research and Education (MVM-CoRE), U.S. Department of Veterans Affairs, Denver, CO 80045, United States of America; VISN 5 Capitol Health Care Network Mental Illness Research Education and Clinical Center (MIRECC), Baltimore, MD 21201, United States of America; Department of Psychiatry, Division of Psychiatric Services Research, University of Maryland School of Medicine, Baltimore, MD 21201, United States of America; VISN 5 Capitol Health Care Network Mental Illness Research Education and Clinical Center (MIRECC), Baltimore, MD 21201, United States of America; Department of Epidemiology and Public Health, University of Maryland School of Medicine, Baltimore, MD 21201, United States of America; Department of Psychiatry, Division of Psychiatric Services Research, University of Maryland School of Medicine, Baltimore, MD 21201, United States of America; Department of Psychiatry, University of Maryland School of Medicine, Baltimore, MD 21201, United States of America; Rocky Mountain Mental Illness Research Education and Clinical Center (MIRECC), U.S. Department of Veterans Affairs, Aurora, CO 80045, United States of America; Military and Veteran Microbiome: Consortium for Research and Education (MVM-CoRE), U.S. Department of Veterans Affairs, Denver, CO 80045, United States of America; Department of Integrative Physiology, Center for Neuroscience, Center for Microbial Exploration, University of Colorado Boulder, Boulder, CO 80309, United States of America; Department of Physical Medicine and Rehabilitation, University of Colorado Anschutz Medical Campus, Aurora, CO 80045, United States of America; Department of Medicine, VAMC Baltimore and University of Maryland School of Medicine, Baltimore, Maryland 21201, United States of America; VISN 5 Capitol Health Care Network Mental Illness Research Education and Clinical Center (MIRECC), Baltimore, MD 21201, United States of America; Department of Psychiatry, Division of Psychiatric Services Research, University of Maryland School of Medicine, Baltimore, MD 21201, United States of America

**Keywords:** statins, lipophilia, hospitalization, schizophrenia, bipolar

## Abstract

**Objective –:**

Psychiatric hospitalizations and emergency department (ED) visits are costly, stigmatizing, and often ineffective. Given the immune and kynurenine activation in bipolar disorder (BD) and schizophrenia, as well as the immune-modulatory effects of statins, we aimed to compare the relative risk (RRs) of psychiatric hospitalizations and ED visits between individuals prescribed lipophilic vs. hydrophilic statins vs. no statins. We hypothesized (a) reduced rates of hospitalization and ER utilization with statins versus no statins and (b) differences in outcomes between statins, as lipophilia increases the capability to penetrate the blood–brain barrier with potentially beneficial neuroimmune, antioxidant, neuroprotective, neurotrophic, and endothelial stabilizing effects, and, in contrast, potentially detrimental decreases in brain cholesterol concentrations leading to serotoninergic dysfunction, changes in membrane lipid composition, thus affecting ion channels and receptors.

**Methods –:**

We used VA service utilization data from October 1, 2010 to September 30, 2015. The RRs for psychiatric hospitalization and ED visits, were estimated using robust Poisson regression analyses. The number of individuals analyzed was 683,129.

**Results –:**

Individuals with schizophrenia and BD who received prescriptions for either lipophilic or hydrophilic statins had a lower RR of psychiatric hospitalization or ED visits relative to nonstatin controls. Hydrophilic statins were significantly associated with lower RRs of psychiatric hospitalization but not of ED visits, compared to lipophilic statins.

**Conclusion –:**

The reduction in psychiatric hospitalizations in statin users (vs. nonusers) should be interpreted cautiously, as it carries a high risk of confounding by indication. While the lower RR of psychiatric hospitalizations in hydrophilic statins relative to the lipophilic statins is relatively bias free, the finding bears replication in a specifically designed study. If replicated, important clinical implications for personalizing statin treatment in patients with mental illness, investigating add-on statins for improved therapeutic control, and mechanistic exploration for identifying new treatment targets are natural next steps.

## 1 Introduction

### 1.1 Psychiatric hospitalizations and emergency room visits

Psychiatric hospitalizations represent markers of instability in severe mental illness and of insufficient therapeutic control, and are economically burdensome and stigmatizing. Even if multifactorial, they are most commonly driven by dangerousness to self or others and psychotic decompensation combined with the lack of social support in the community [1]. Inpatient hospitalization often fails to fulfill its main rationale, suicide risk management, as the discharge from the inpatient unit brings with it a substantially elevated potential for suicidal behavior [2,3]. In addition to psychiatric inpatient hospitalization, the use of Psychiatry Emergency Departments (ED) in the United States (US) is responsible for substantially increasing costs of health care [4,5] and diverting funds from services that would be expected to have a potentially more impactful contribution to quality care and preventative services. This is particularly true for the care of US Veterans, considering that the Veterans Health Administration (VHA) serves a population with considerable mental health needs [6] and has steeply increasing costs [7]. The top 10% of utilizers of psychiatric inpatient [8] and emergency services [9] are responsible for a disproportionally high share of healthcare costs. In the VHA system, understanding the clinical, social, and economic factors driving the utilization of psychiatric emergency services has relied on the availability and the analysis of administrative data [10,11] and has identified severe mental illness, personality disorders, substance use disorders, utilization of detox services, and homelessness as dominant predictive variables [12–14]. All in all, it is common knowledge for anyone who either worked or studied psychiatric emergency services that the reason for psychiatric hospitalization is the evaluation and management of acute suicide risk [2]. Thus, to a certain degree, psychiatric hospitalization also serves as a proxy measure of suicide risk. While important everywhere, in US Veterans, in particular, death by suicide and its prevention are the indisputable #1 priority. According to the latest available yearly report (2020), the 2018 age- and gender-adjusted rate of suicide in US Veterans was 27.5 per 100,000, 50% higher than in the US general population, as it has been each year since 2013–2014 [15]. In Veterans with mental health or substance use disorders, the 2018 rate was significantly twice, at 57.2 per 100,000. More than 17 Veterans are prematurely lost to suicide each day, with guns being the lethal means for 41.9% of female Veteran suicides and 69.4% of male Veteran suicide deaths.

### 1.2 Medications for medical conditions affect psychiatric condition

Comorbidity with medical illness often leads to worsening of the course of mental illness. However, there is a potential of the medical treatment per se to worsen mental health, directly (e.g., systemic corticosteroids decompensating mood disorders) or through interactions with psychiatric medication (e.g., thiazide diuretics for hypertension dramatically increasing lithium serum levels and causing toxicity in patients with bipolar disorder, or the commonly prescribed seizure medication phenytoin lowering the levels of clozapine in patients with schizophrenia and inducing a recurrence or exacerbation of psychosis).

Yet, there are circumstances when a specific medical treatment could have a beneficial effect on mental health. Examples include beta-blockers for anxiety, certain calcium blockers as mood stabilizers, certain alpha-adrenergic blockers on nightmares (prazosin), and others.

### 1.3 Statins

#### 1.3.1 Pleiotropic effects

Statins, one of the most prescribed groups of medications worldwide, have been increasingly recognized as having therapeutic value for a variety of conditions beyond their original metabolic and cardiovascular indication [16]. Early, when the main data were anecdotal and observational, there were concerns that statin may worsen mood and cognition and have a negative impact on neuropsychiatric conditions and potentially elevate the suicide risk via the association between very low levels of serum cholesterol and suicide. With the emergence of case-control, longitudinal, and especially randomized controlled studies and their meta-analyses, the opposite picture emerged, i.e., that statins as a group are cognitively, affectively, and behaviorally safe and that they show beneficial effects in the treatment of mood disorders, schizophrenia, and cognitive disorders. These positive effects appear to be based on statins’ pleiotropic effects including multiple immune regulatory actions and antioxidant properties [17,18], as well as endothelial stabilizing [19] and neuroprotective effects [20,21]. Statins reduce NADPH oxidase and superoxide generation, inhibit the negative regulation of nitric oxide, increase free radical scavenging, decrease inflammatory cell transmigration from blood to tissue, inhibit the NLRP3 inflammasome, and reduce metalloproteinase expression [22–27]. Notably, evidence also suggests that statins facilitate PI3K-Akt signaling [28,29] and crosstalk with peroxisome proliferator-activated receptor (PPAR) signaling [30]. In addition, within the brain, statins are potent inducers of axonal and neurite outgrowth; in fact, among more than 50,000 small molecules previously implicated in axonal outgrowth, statins were the most effective [31]. Activation of the Akt signal transduction and RhoA prenylation underlie the statin-dependent neurite outgrowthn, which may account for the effects of statins to promote structural repair of injury and to reduce excitotoxicity, leading to the establishment, and maintenance of short- and long-distance connectivity with following injury. Consistently, high-dose simvastatin, a highly lipophilic statin, leads to less brain atrophy than a placebo-control group using serial volumetric MRIs in patients with the prototypical autoimmune disease of the brain-multiple sclerosis (MS).

#### 1.3.2 Statins are beneficial for medical conditions characterized by inflammation

Statins have favorable long-term effects in individuals with medical conditions characterized by increased inflammation, both in medical conditions with increased inflammation primarily within the brain and in medical conditions with increased inflammation primarily in the periphery. For instance, sustained statin pretreatment and continuation lead to decreased mortality following traumatic brain injury (TBI; a condition in which inflammation plays a strong dual role of providing surveillance and removal of necrotic tissue, and also, being a negative prognostic indicator and a local mediator of pathophysiology leading to prolonged symptomatology, delayed healing, and limited functional recovery), as well as a more rapid hospitalization discharge, decreased depression post-TBI, and improved functional recovery at 12 months post-injury [32]. In patients with allergies, statin use is associated with a decreased risk of asthma-related ED visits [33,34] and/or hospitalizations [33]. Statins have also demonstrated benefit across autoimmune conditions, particularly, in MS [35].

#### 1.3.3 Inflammation and immune-mediated conditions – links with mental illness and suicidal behavior

The involvement of inflammation in mental illness [36], including schizophrenia [37–40], bipolar disorder [41], and suicidal behavior [42–45], has been increasingly recognized. For instance, individuals with schizophrenia have a peripheral blood elevation of interleukin (IL) 6, IL-1β, and transforming growth factor-β1 (TFG-β1) [28], and, centrally, have molecular and visual markers of microglia activation relative to healthy controls [29], leading to ongoing vulnerability to immunological challenges and psychological stress. Immune-mediated clinical conditions implicated in schizophrenia have included infections, autoimmune disease, and atopy [46]. Similarly, suicidal behavior – a common driver of ED visits and hospitalizations – has also been associated with infections, with either specific microbial agents, such as *Toxoplasma gondii*, [47–51] or nonspecific microbial agents [52,53], allergy [54], and allergen exposure [55–57]. As illustrated in both preclinical and clinical studies, statins manifest a potent antimicrobial action across a broad spectrum of intracellular pathogens, including viruses, bacteria, protozoa, and fungi, interfering with the host mevalonate pathways and compromising the microbial immune evasion. Statin administration prolongs survival in certain infectious diseases by protecting from the overly intense and prolonged immune response, in addition to promoting host defense [58].

#### 1.3.4 Statins in depression

Given the considerable overlap among elements of the immune, oxidative stress, excitotoxic, endothelial, and immune regulatory dysfunction in severe mental illness and reversal of these effects with statins, promising reports on experimental/animal models and early clinical data using statins for augmentation of classical psychotropic agents became increasingly attractive. While heterogenous and far from definitive, increasingly compelling data exist in support of with distinct clinical and physiopathological benefits with of the use of statins; with increasingly sophisticated and well-sized studies, the earlier concerns about potentially major negative effects of statins are slowly receding. For example, awareness of successful augmentation of antidepressant effects in major depression (MD) by statins begins to emerge, starting with a small meta-analysis [59] (3 studies on 165 participants), followed by a meta-analysis of 36 randomized placebo-controlled studies of anti-inflammatory add-on interventions including 7 randomized placebo-controlled studies on 1,576 participants [60]. This was further confirmed by a large meta-analysis of multiple anti-inflammatory approaches by Bai et al., including 30 studies, and 1,610 participants, including 3 randomized controlled trials of statins with 166 participants [61]. In the most recent meta-analysis of randomized clinical trials on 5 studies and 389 participants, De Giorgi et al. reported significant benefits of add-on statins at 8 and 12 weeks of treatment. In comparing statins, simvastatin, the most lipophilic compound, demonstrated a stronger antidepressant capability than atorvastatin [62]. This was consistent with a previous study on the antidepressant effect of statin in post coronary artery bypass graft with mild-moderate clinical depression, demonstrating a greater efficacy of simvastatin over atorvastatin [63].

#### 1.3.5 Statins in schizophrenia

In schizophrenia, a meta-analysis of 5 RCTs (with 236 adult participants with schizophrenia, neuroleptic treated) found improvement in positive and negative symptom scores with an add-on statin vs. placebo [64]. A larger meta-analysis (70 studies, 4,104 participants) also including other potential anti-inflammatory add-on interventions confirmed a significant, yet small, improvement in positive and negative symptom scores (7.1%) with statins relative to placebo [65]. In contrast, another meta-analysis (56 studies, 4,327 participants) found no significant benefit from an add-on statin [66]. Shen et al. [67] embarked on an ambitious meta-analysis not only to evaluate if statins improved negative or positive symptoms but also to see if different classes of statins have differential effects, if different types of antipsychotics combined with statins had a different effect, and finally, to measure the effect of the duration of treatment with statins. At week 12, a significant difference emerged in both positive and negative symptoms between the statin antipsychotic group vs. the placebo antipsychotic group. In terms of differences between statins, simvastatin manifested the strongest effect for both positive and negative symptoms, and it was the routine antipsychotics, rather than the new-generation antipsychotics, that showed improvement in both positive and negative symptoms [67]. The authors explained their finding in terms of both statins (simvastatin [68], atorvastatin, rosuvastatin, pravastatin, and lovastatin [69,70]), and antipsychotics (strongest with risperidone [and quetiapine] and weakest for haloperidol and clozapine) being P-gp substrates of P-glycoprotein 1 (P-gp), also called ATP-binding cassette sub-family B member 1 (ABCB1). Antipsychotic agents interact with statins through competition for P-gp transport. Being co-substrates, the lower affinity antipsychotics and statins will result in reciprocal greater access to the CNS; the results indeed supported a considerable component of the effect of statins on negative symptoms to be the result of interactions between statins and the type of the antipsychotic.

What could explain the beneficial effect of statins in schizophrenia reported by the majority of the meta-analyses published to date, in particular in regard to domains that are so resilient to neuroleptic treatment, such as negative symptoms and cognitive deficits? Indeed, negative symptoms and cognitive deficits in schizophrenia are the two symptomatic domains least responsive to treatment and most contributory to functional deficits in individuals with the illness. Immune activation has been increasingly identified in individuals with schizophrenia, and, in particular, proinflammatory signals are associated with the severity of cognitive dysfunction and negative symptoms [71,72] with likely disruptive effects on synaptic signaling, neurogenesis, neuroprotection, axonal/neurite growth, and thus connectivity [73]. In addition, oxidative stress, in part a consequence of a proinflammatory and excitotoxic milieu, disrupts parvalbumin interneurons. The disruption of these interneurons localized in the cortex and hippocampus, known to have an increased vulnerability to oxidative stress, may centrally contribute to the production of cognitive dysfunction and negative symptoms [74]. In addition, executive dysfunction in schizophrenia is also, in part, a consequence of increased oxidative stress, as illustrated peripherally by several molecular systems, such as glutathione/GSH and, centrally, via reduced neurotrophic factors, such as brain-derived neurotrophic factor (BDNF) [75]. All these targets, the oxidative stress, neuroinflammation, neuroprotective and neurotrophic deficits, and molecular inflammation cascades are engaged and modulated by statins [76,77].

#### 1.3.6 Statins in bipolar disorder

Similarly, individuals with bipolar disorder also have clinical and preclinical evidence for immune dysregulation, with the elevation of proinflammatory cytokines relative to psychiatric and normal controls, including IL-6, tumor necrosis factor (TNF)-α, IL-1β, soluble receptor of TNF-type 1 (STNFR1), and soluble interleukin-2 receptor (sIL-2R) [78–84]. Isolated reports have been confirmed in a recent systematic review/meta-analysis that documented a “traitlike” elevation of proinflammatory signals during both mania and depression episodes, reverting to normal values after return to euthymia [85]. Of functional importance, the markers of immune activation are positively associated with cognitive status, as well as neuroanatomical changes [86–90]. Yet not all studies have found evidence of neuroinflammation in BD [91]. In contrast with the classical view of bipolar disorder being in principle an episodic condition with full restoration to normality, there is evidence that residual mood and cognitive symptoms, functional impairment, decreased quality of life, and psychosocial disability exist even when the condition is appropriately treated [92,93]. Moreover, there is evidence for bipolar disorder being a progressive condition, often in the context of nonadherence and inappropriate treatment resulting in highly recurrent mood episodes, incomplete interepisodic remission, progressive cognitive impairment, and functional decline [94–96], with specific neurobiological underpinnings characterizing different stages and longitudinal progression of bipolar disorder (“neuroprogression”) [97–102]. Of relevance for possible treatment targeting by statins, neuroprogression has been linked to enhanced oxidative stress, breakdown of the neurotrophic support, mitochondrial dysfunction, and decreased cellular resilience, in part as a result of persistent and cascading immune dysfunction [99,100,103,104], leading to the loss of neuroprotection, excitotoxicity, apoptosis, and loss of cortical volume. Yet not all studies agree with the neuroprogression hypothesis in bipolar disorder, in particular with the accelerated cognitive decline with aging [100,105].

### 1.4 The tryptophan degradation and the kynurenine pathway

Immune activation and stress lead to activation of enzymatic systems such as indoleamine 2,3-dioxygenase (IDO) or tryptophan 2,3-dioxygenase (TDO), resulting in increased production of kynurenines and decreased tryptophan [106]. Mitigating potential pathogenic effects of infection, the decrease of tryptophan affects multiplication of microbes, preventing unchecked invasion of the organism, and kynurenine production is immune regulatory, preventing unremitting inflammation. Kynurenine can cross the blood-brain barrier (BBB) freely and is degraded to molecules produced locally without the capability to freely cross the BBB. These have excitotoxic effects (e.g., quinolinic acid [QA]) or inhibitory effects (kynurenic acid [KA]) on brain structure or function. Suicidal behavior is associated with elevations in kynurenine [107] and QA [108,109], while schizophrenia [110,111] and BD with history of psychotic episodes [112] are associated with with elevations of KA. More recently, positive associations between cognitive deficits in schizophrenia and QA in schizophrenia have been reported, suggesting a state of excitotoxic necrosis as the basis of these symptoms, highly relevant for functioning, rehabilitation, and prevention of suicidal behavior in schizophrenia [113]. Dysregulation of the kynurenine pathway has also been implicated in BD and its progression [114], as well as state severity of depression in patients with bipolar disorder [115].

### 1.5 Statins: Anti-inflammatory and immune regulatory effects

Statins generally decrease, in a concentration-dependent manner, the production of neopterin and degradation of tryptophan in *ex vivo* models, such as peripheral blood mononuclear cells (PBMCs) stimulated by IFN-gamma, concanavalin A (ConA), and phytohemagglutinin (PHA) [116] . In patients with suspected stable angina pectoris who are taking statin therapy after angiography, increased neopterin level is associated with the increased risk of acute myocardial infarction (AMI) [117]. Consistent with these findings, in patients with acute coronary syndrome (ACS), higher neopterin levels predict the increased long-term risk of death and nonfatal coronary events. In these patients, high-dose statin treatment reduces neopterin levels, while decreasing coronary mortality and nonfatal coronary events [118] . Statins inhibit the cellular proliferation of PBMCs induced by different antigens, such as T-B polyclonal stimuli and *Staphylococcus aureus* enterotoxin A (SEA) [119].

However, not all studies have concordant results. For instance, in one clinical longitudinal study, statins led to decreased C-reactive protein (CRP) levels, but had no effect on neopterin levels and autoantibody titers (such as antinuclear antibodies) [120]. Furthermore, a double-blind placebo-controlled randomized trial of statin administration found no significant change in inflammation biomarkers (neopterin, CRP) [121].

Statins also manifest immune-modulatory effects by activating regulatory T cells (Tregs). For example, in a murine model of tumor growth, statins activate Treg and increase the production of the immune regulatory markers IL-10 and TGF-β1 [122]. Statins reduce the number and increase the suppressive function of Treg cells in animal experimental models of chronic immune activation [123] and in humans with or without immune-mediated conditions [124]. The production of Tregs is linked with the production of reactive oxygen species (ROS) geared at a minimum toward eliminating pathogens, at most, to regulate the balance between proinflammatory and regulatory arms of the immune response [125]. This has a narrow regulatory window as specifically unopposed ROS production can suppress regulatory T cell production in favor of proinflammatory reactions, or alternatively, very little ROS production may also impair the differentiation, stability, and suppressive function of Tregs [125].

Statins not only reduce the production of kynurenine but also actively limit the effects of kynurenine’s main excitotoxic metabolite, QA. For example, in a model of QA-induced neurotoxicity in rats, statins significantly decrease the excitotoxic effect of QA, levels of markers of oxidative stress, and proinflammatory cytokines (such as TNF-α) as well as striatal lesion volume [126]. In another study, statins appear to have a neuroprotective effect in excitotoxic rodent seizure models. Specifically, atorvastatin significantly reduced QA-induced clonic and/or tonic seizures and prevented cell death induced by QA in the hippocampus [127]. Atorvastatin also counteracts the decrease in glutamate uptake triggered by QA and prevents the QA-induced decrease in protein kinase B (PKB, or Akt) phosphorylation [128].

### 1.6 Lipophilic vs. hydrophilic statins

Statins are characterized by different degrees of lipophilicity and divided into lipophilic and hydrophilic categories. While both groups have a similar efficacy in reducing cardiovascular and general mortality and overall side effects [129], the lipophilic statins cross more readily the BBB [130] and thus raise the hope that they could have a more pronounced local anti-inflammatory, antioxidant, neuroprotective, and endothelial-stabilizing effect within the CNS, and lead to improvement in conditions with clear primary brain localization of pathological processes. On the other hand, their potential toxic effects on cellular lipid components of membranes in affecting transmembrane receptors and channels, and other deleterious molecular effects within the brain could offset, at least partially, the beneficial CNS effects of statins [76,77].

### 1.7 Rationale and hypotheses

The increasing understanding of immune dysregulation across diagnostic categories of mental illness, including MD, BD, schizophrenia, and dementias (of Alzheimer’s type, vascular type), has been the number one rationale to consider using add-on statins, especially in individuals who already have medical indications. Indeed, several meta-analyses, increasingly including randomized clinical trials (RCT), support the clinically beneficial effects of add-on statins in depression and schizophrenia, although meta-analytic and RCT exceptions exist. Similarly, statins appear to have been beneficial in cognitive disorders by slowing cognitive decline [131], reducing the risk of dementia [132], improving Mini-Mental scores, and slowing deterioration on neuropsychiatric inventory deficit scores[133].

A large population-based study on Danish nationals with MD treated with selective serotonin reuptake inhibitors (SSRIs) found a reduced hazard of psychiatric hospitalizations with concurrent statin use compared to a no statin treatment group (hazard ratio, 0.75; 0.64–0.75) [134]. To our knowledge, there is no equivalent study on hospital contacts in schizophrenia and BD. We now aimed to fill this gap. We went one step further: Because lipophilic statins and hydrophilic statins differ in terms of their ability to cross the BBB (specifically lipophilic statins being able to cross more readily the BBB, thus having effects also on the CNS, and not only peripherally, as the hydrophilic statins), we engaged the aim of comparing the two statin categories on their ability to prevent psychiatric hospitalizations and ED visits when added on to antipsychotic treatment (in schizophrenia and bipolar individuals) and mood stabilizer treatment (in bipolar individuals). In doing so, we intended to minimize confounding by indication and to take a first step in the direction of personalizing the choice of statins for individuals with mental health problems. We used VA administrative data on ER visits and psychiatric hospitalizations as well as VA pharmacy data. We hypothesized that add-on statins will be protective relative to no statin control and that differences between lipophilic and hydrophilic statins will emerge (two-tailed hypothesis). Psychiatric hospitalization and ED visits were pair-wise compared among the three statin prescription groups (lipophilic, hydrophilic, and none) separately in antipsychotic-treated individuals with schizophrenia, as well as antipsychotic - or mood stabilizer-treated individuals with BD, using robust Poisson regressions.

## 2 Methods

### 2.1 Overall design

Overall, this is a hypothesis-testing observational study using VA health care service utilization data from October 1, 2010 to September 30, 2015, including demographic, diagnostic, hospitalization, and ED visits, and all outpatient prescription medications dispensed from VA pharmacies, as reported in a recent publication using a similar methodology with the same dataset [135].

### 2.2 Data sources

This study used data on health services maintained at the U.S. Department of Veterans Affairs’ (VA) Corporate Data Warehouse (CDW). The study period was between October 1, 2010 and September 30, 2015. All healthcare inpatient and outpatient workloads provided to Veterans in VA hospitals and outpatient clinics across the US were included. Hospitalizations that occurred in non-VA hospitals were included via the VA fee base files.

#### Ethical approval:

This study was approved by the Institutional Review Board of the University of Maryland School of Medicine. The conducted research is not related to either human or animal use.

#### Informed consent:

Institutional Review Board of the University of Maryland School of Medicine approved a waiver of informed consent because we used only extant administrative data.

### 2.3 Sample selection

We selected all VA health records, which had, during the study period, at least one outpatient or inpatient ICD-9- CM code for schizophrenia (295×) or BD (code disorder (ICD-9-CM code of 296.0–1, 296.4–8). If other serious mental illness codes (296.×, 297.×, 298.×) were present, we assigned the diagnosis that was present during the majority of encounters in the study period. A total of 683,129 participants were included in this study. Among them, 185,449 were individuals with schizophrenia treated with antipsychotics, 211,412 were individuals with BD treated with antipsychotics and 286,268 were patients with BD treated with mood stabilizers.

### 2.4 Medications

Incident treatment episodes were constructed from prescription records. Lipophilic statins included prescriptions for simvastatin, atorvastatin, pitavastatin, and lovastatin. The hydrophilic statins included rosuvastatin, pravastatin, and fluvastatin. A total of 69.69% of individuals in the entire population during the duration of the study had at least one statin prescription dispensed at a VA pharmacy, with most commonly dispensed lipophilic statins being simvastatin (in 33.16% of individuals) followed by atorvastatin (18.07%), hydrophilic statins pravastatin (9.49%), and rosuvastatin (7.76%). Lovastatin, pitavastatin, and fluvastatin were seldomly dispensed (combined less than 2%). A treatment episode was defined as a continuous time interval of medication possession from the specific date of the incident prescription until the first prescribing time gap between prescriptions of more than 15 days. Gaps of at least 16 days past the expected refill date were considered clinically significant and consequently were interpreted as a discontinuation of a prescribed medication. Imperfect adherence was defined as gaps of 2 weeks or less, was considered clinically irrelevant and was ignored.

### 2.5 Outcomes

To help identify the average effect of each medication, we examined only incident prescribing episodes, defined here as no prescriptions for the same medication in the 6 months before the designated episode start date. To ensure sufficient numbers of individuals prescribed a medication with an adequate exposure period were available for analysis, we retained only those medications that had at least 100 individuals with schizophrenia or BD with episodes lasting at least 3 months. To facilitate the identification of episodes lasting at least 3 months, we limited analyses to episodes with start dates between April 1, 2011, and March 31, 2015.

### 2.6 Comparisons

We examined the relative risk (RR) of psychiatric hospitalization and ED visit during incident prescription episodes comparing lipophilic to hydrophilic statins, and also each statin group to a “no statin” control group. In each of the three analyses by diagnosis-psychiatric medication, the control group (no statin – “none”) consisted of all other nonpsychiatric incident medication episodes – excluding those for hydrophilic or lipophilic statins – that lasted 3 or more months for at least 100 individuals.

We only included patients who received a prescription of primary psychotropic medication during the 6 months before the start of the prescription episode. Individuals with schizophrenia were excluded if they did not have an antipsychotic prescription in the 6 months before the start of any episode, and individuals with BD were excluded if they were not on a mood stabilizer or antipsychotic during this period. As such, we performed three separate analyses for each outcome – one for patients with schizophrenia treated with antipsychotics, one for BD patients treated with antipsychotics, and one for BD patients treated with mood stabilizers.

### 2.7 Statistical analyses

We analyzed the data with the Statistical Analysis System (SAS) Version 9.4, Cary, NC. Robust Poisson regression [136] was used to estimate the RR of psychiatric hospitalization and ED visits comparing incident episodes of lipophilic vs. hydrophilic statin use and each statin vs. the “other” control group episodes. Robust Poisson regression provides accurate standard error estimates when the outcome is binary by using robust (“sandwich”) standard errors. The robust standard errors also account for within-individual correlation among repeated prescription episodes. In those with schizophrenia and those with BD on antipsychotic medication, we adjusted for age, gender, race, ever married, prior psychiatric hospitalization or ED visits, posttraumatic stress disorder (y/n), alcohol use disorder (y/n), other substance use disorder (y/n), antipsychotic coverage, mood stabilizer (y/n), and antidepressant (y/n) in the prior 6 months. In the group of bipolar individuals on mood stabilizers, all the covariates were identical, with the exception of antipsychotic coverage being replaced with antipsychotic (y/n) and mood stabilizer (y/n) being replaced with mood stabilizer coverage. A parallel analysis was performed to estimate RR of ED visits in the 6 months after the prescription episode began.

## 3 Results

### 3.1 Descriptive results

The participants with schizophrenia with antipsychotic medication had the mean (SD) age of 58.67 (9.99) years. The majority of these participants were men (91.66%) and white (60.13%; Table 1). The participants with BD on antipsychotic medication had the mean age (SD) of 54.77 (11.29) years. The majority of these participants were men (82.35%) and white (80.18%; Table 2). Their demographic composition was very similar to that of the bipolar participants on a mood stabilizer (Table 3).

### 3.2 Hypothesis testing analysis

The RR of psychiatric hospitalization was higher in those prescribed lipophilic statins in comparison to those prescribed hydrophilic statins in all three diagnosis X treatment groups, in individuals with schizophrenia on antipsychotic medication (RR = 1.11; CI, 1.0007–1.23; *p* = 0.048), in individuals with BD on antipsychotic medication (RR = 1.22; CI, 1.09–1.36; *p* = 0.001), and in individuals with BD on mood stabilizers (RR = 1.18; CI, 1.06–1.32; *p* = 0.002; Table 4). The RR of psychiatric hospitalization was lower in those prescribed either statin (lipophilic vs. hydrophilic) than in the control “no statin” group (Table 4).

After adjustment, there were no statistical differences in the RR for ED visits between lipophilic and hydrophilic statins in all three diagnosis X treatment groups, in individuals with schizophrenia on antipsychotic medication (RR = 1.07; CI, 0.83–1.36; *p* = 0.61), in individuals with BD on antipsychotic medication (RR = 1.26; CI, 0.97–1.63; *p* = 0.08), and in individuals with BD on mood stabilizers (RR = 1.16; CI, 0.92–1.46; *p* = 0.2). The differences between each statin group and control “no statin” group were robust, with lower RR of ER visits in those on either statin group relative to controls (see Table 5).

## 4 Discussion

### 4.1 Key results in context

The add-on of either lipophilic or hydrophilic statin prescription to US Veterans diagnosed with and treated for BD or schizophrenia was associated with a lower RR of psychiatric hospitalizations or ED visits as previously reported for patients with MD treated with SSRIs [134]. The add-on hydrophilic statins prescribed to individuals with BD and schizophrenia had a lower RR of psychiatric hospitalizations, but not of ED visits, than the lipophilic statin prescription in the same clinical groups. Although no similar comparisons between lipophilic and hydrophilic statins have been undertaken, to our knowledge, the results of a protective effect of statin use on hospitalization rates were consistent with one study nested in Danish registers showing that statin (simvastatin) treated patients with citalopram had fewer hospital contact than patients treated with SSRI alone [137]. The results are also consistent with a military study, revealing the increased risk of developing schizophrenia symptoms in nonpersistent statin users vs. persistent statin users [131]. It is important to keep in mind that the majority of studies of positive effects of statins on depression, schizophrenia, and on a delay of Alzheimer’s disease onset were based on lipophilic statins, and an elevated risk of suicidal ideation with statin (a common reason for psychiatric hospitalizations) has been also reported in lipophilic statin use only [138]. Yet direct comparisons between two statin categories have seldomly, if at all, been undertaken. Mild cognitive impairment [139,140] and sleep disturbances [141,142], both a consequence as well as a possible contributor to poor therapeutic control, occurred more commonly with lipophilic rather than hydrophilic statin administration, suggesting that brain penetrance may engage mechanisms that could offset, in part, anti-inflammatory, antioxidant, and endothelial positive effects of statins, perhaps via lowering cholesterol in the neuronal membranes and impairing channel and receptor function and/or mitochondrial function. However, this has to be interpreted with skepticism, as recent meta-analyses have not found any significant cognitive impairment of statin use with mild cognitive deficits [143,144] or with sleep efficiency or duration [145].

### 4.2 Confounding by indication

In comparisons between statins and no statins, there is a likely confounding by indication based on comorbidity with metabolic and cardiovascular conditions that may affect the response to medication, alter the risk of self-harm, and alter the risk of psychiatric hospitalization and ED visits. Methods to weigh, balance, or adjust for potential sources of bias, e.g., propensity score-matched analyses as in the supplemental material presented in the study by Kohler et al. [134], are deemed to remain incomplete without including the specific indications for statin treatment, such as low-density lipoprotein and total cholesterol measurement. Thus, our current comparison between statins and no statins, as Kohler’s finding in MD, should be received with considerable skepticism. Yet the comparison of lipophilic and hydrophilic statin minimizes bias [16] and may lead to an important decision point in those individuals who have schizophrenia or BD and meet the criteria to initiate or restart the statin treatment.

### 4.3 Statin effects on cognition and cognitive disorders

Cognitive deficits, as well as negative symptoms, are strongly predictive of functioning and course in schizophrenia and bipolar disorder and, although to a limited degree, are modifiable with add-on statin treatment [76,77]. Theoretically, statins could be particularly promising for cognitive deficits in individuals with schizophrenia and bipolar disorder as they have been reported by some (but not all) studies on cognitive deficits in the context of aging (age-related cognitive decline) and dementia. For instance, in community-dwelling adults, slower cognitive decline [131] and fewer white matter abnormalities [146] have been associated with statin treatment.

Wong et al. (2013) performed a systematic review of 20 uncontrolled studies on 4 million participants and identified a mild beneficial effect in Alzheimer’s dementia and all dementias. Similarly, Song et al. (2013), via a meta-analysis of 11 longitudinal cohort studies and 57,020 participants, with positive heterogeneity and no evidence of bias, identified an approximately 40% decreased risk of dementia with the use of statins [132]. At the same time, Swiger et al. found in a meta-analysis of 11 studies with 23,443 individuals [144] enrolled in RCTs and prospective cohort studies identified a 29% protective effect of statins at the long term (>1 year), while there were no immediate benefits at a short term (<1 year). Most recently, the meta-analysis of Xuan et al (2020) on exclusively RCTs (nine studies) and 1,489 participants, uncovered a statistical improvement in cognitive function by Mini-Mental score and a reduced progression of worsening based on the Neuropsychiatric Inventory Questionnaire, but with no differences in other measures [133].

In a recent large study, bipolar individuals using neurocognitive testing belonging to four cognitive domains, statin users and nonusers did not differ in regard to cognitive function [15]. The authors concluded that statins neither improve nor deteriorate cognitive functioning in bipolar patients [147]. They suggested that inherent cognitive deterioration secondary to bipolar disorder, i.e., “neuroprogression” [105,148], and aging per se [100] outweigh the beneficial effects of statin treatment.

Statins have been linked also with mild cognitive impairment [149–151], findings that stand in full opposition to those reporting a delayed decline in cognitive function in older adults [149–151], with several studies that found no significant association [143,144]. Of relevance and congruent with our current report, clinical trials of hydrophilic statins did not elicit any cognitive deficits [76], while lipophilic studies were associated with mild cognitive impairment [139,140].

### 4.4 Biological mechanisms potentially contributing to our key results

#### 4.4.1 Immune modulatory effects

Statins increase the number and increase the suppressive function of Treg cells in animal experimental models of chronic immune activation [123], clinically both in the presence and in the absence of immune-mediated conditions [124]. In addition, an increasing number of randomized controlled studies and meta-analyses support substantial benefits of statins as add-on interventions in various mental health conditions. The benefits of statins in mental health have been attributed to several molecular, cellular, and system effects, including modulating inflammation and endothelial function and reducing oxidative stress [18]. In particular, for the diagnostic categories analyzed in this project, the immunoregulatory effects of statins specifically engage and modulate prion-flammatory targets associated with cognitive dysfunction and negative symptoms [71,72], key determinants of functioning in schizophrenia. In addition, statins have been reported to modulate molecular and cellular signatures involved in the trait-like immune activation during mania and depression in BD [85] as well as the progressive immune dysfunction [99,100,103,104] linked with the cognitive and functional decline in “neuroprogressive” cases of BD [97–102]. Most importantly, in conditions of acute neuro-adversity known to be downstream mediated by systemic inflammation and oxidative stress, statins have an overall immunoregulatory effect. For instance, in a murine model of TBI, atorvastatin significantly increases the proportion of regulatory T cells (Tregs) in both the periphery (spleen) and the brain and simultaneously increases their main immunoregulatory effectors - anti-inflammatory cytokines such as IL-10 and TGF-β1 [152], with the reduction in histopathology and functional recovery.

#### 4.4.2 Antimicrobial effects

Statins are also potent add-on antimicrobial agents by interfering with mechanisms that pathogens use to evade the immune response and preventing immune overactivation and unremitting activation, which might have an important role in preventing reactivation of latent infections, such as chronic toxoplasmosis, implicated in schizophrenia [153] or suicidal behavior in individuals with schizophrenia [51] or with recurrent mood disorders [49,154].

#### 4.4.3 Potential mechanisms for lipophilic vs. hydrophilic divergence

What mechanisms could underlie the difference in outcome between the lipophilic and hydrophilic statins? First, cognitive dysfunction may be elevated in the group of statins with penetrance in the brain, potentially through affecting neurotransmitter receptors, ion channels (via changes in the cholesterol component of lipid membranes), or, as in Alzheimer’s Dementia, induction of axonal degeneration due to deprivation of cholesterol as shown in cultured neurons [155]. Furthermore, a much more intense lowering of brain cholesterol by the lipophilic statins - with no cholesterol intake possible from outside the CNS - may affect brain serotonin synthesis, thus impulsivity, aggression, anxiety, violent ideation, and thus increased risk for self-directed violence [156–158], and, in response, ED visits and psychiatric hospitalizations.

In addition, another interactive effect is possible, i.e., local intense antioxidant effect by lipophilic statins within the CNS may adversely affect the TH_1_ immune pressure on intracellular pathogens such as CMV or *Toxoplasma gondii*, which may result in reactivation of the pathogens and loss of therapeutic control. Furthermore, the plunging of brain ROS levels could also affect the maturation and function of Tregs, with reduced mediation of immunomodulatory signals (such as IL-10) and overshooting of inflammatory reactions promoting, among other mechanisms, persistent microglia hyperreactivity. For instance, in cell cultures, TNF-α upregulation and microglia activation [159,160] and induction of the mitochondrial apoptotic pathways, time and dose-dependent, have been reported with lovastatin [161].

#### 4.4.4 Panorgan endothelial-stabilizing effects of statins

Furthermore, the effects of statins on stabilizing endothelial function [19] could be, in conjunction with their peripheral anti-inflammatory effect, the most important mechanisms for symptomatic, functional, and behavioral effects of statins, and thus, the effect on brain and behavior would primarily take place via diffuse vascular mechanisms. This has been best documented for rosuvastatin, the most commonly prescribed hydrophilic statin in our sample, given its potency and vascular tropism, and devoid of the added toxicity of penetration of brain parenchyma with lipophilic statins, such as the toxic effects on brain mitochondrial pathways.

Several molecular underexplored mechanisms could underlie the effects of statins. As autophagy-related pathway impairment has been reported in schizophrenia [162], it is possible that statin-mediated neuroprotection is based, at least in part, on restored autophagic mechanisms as robustly reported for rosuvastatin (the most prescribed hydrophilic statin) in Parkinson’s disease [163].

#### 4.4.5 Statin kinetic interactions with psychotropic medications

First, coupling between a specific psychotropic agent and a specific statin may lead to interactions at the level of the cytochrome system involved in their metabolism resulting in blood level changes on the psychotropic, or of the statin. For instance, fluvastatin (lipophilic) and rosuvastatin are metabolized via CYP 2C9 and all others (with the exception of pravastatin) via CYP 3A4 [164]. Pravastatin is eliminated renally with minimal metabolism at the CYP system [165]. For example, the mood stabilizer carbamazepine is a strong inducer of the CYP 3A4 and thus is expected decrease the blood level of most statins, bringing it below the high-dose requirement that appears clinically necessary for effectively augmenting psychotropic medications or protecting cognitive function. On the other hand, the SSRI fluvoxamine, used almost exclusively in obsessive compulsive disorder (OCD), a condition often comorbid with schizophrenia, is a potent inhibitor of CYP 3A4, and thus resulting in considerable increases in levels for the majority of statins (thus meeting the high-dose requirements established for positive psychiatric effects based on early clinical impression, or, alternatively, contributing to toxicity and statin discontinuation). This combination may put a patient at a major risk for myopathy, liver and kidney toxicity, situations that for many patients practically represent an early end of statin treatment effects based on early clinical impression. Other potential interactions between statins and antipsychotics are based on competition for P-glycoprotein 1 (a BBB transporter guarding brain molecular access) and, being both substrates, act additively or synergistically for achieving a higher concentration in the CNS, leading to increased efficacy or amplified side effects [76]. This interaction is likely statin and antipsychotic specific based on specific P-glyco-protein 1 affinity (e.g., high for risperidone and low for clozapine or haloperidol) and, with advanced knowledge, amenable to deliberate statin by psychotropic coupling. These effects and specific interactions should be taken into considerations in future studies using machine learning to weigh potential effect modifiers and confounders among statin groups, individual statins, and statin/psychotropic combinations to identify sources of heterogeneity in clinical benefits.

#### 4.4.6 Strength

We utilized a systematic data mining approach and yet a focused hypothesis-driven stance. The lipophilic vs. hydrophilic comparison is based on a dichotomy, central vs. peripheral effects, and known capability to cross the BBB. This comparison minimizes confounding and reduces bias.

#### 4.4.7 Limitations

The generalizability might be relatively limited. We analyzed prescriptions (i.e., picking up the prescription) and not adherence based on actually taking the medications. We have not taken into consideration the relative potency of statins, the duration of administration, degree of adherence, and dosage (e.g., by accounting for a “dose-equivalent” across specific statins). The few studies that considered these variables suggested advantages for long duration and high dosages [166,167].

Time-varying confounders, such as interactions with psychiatric medications, have not been analyzed, and time-invariant confounders such as genetic factors and lifestyle characteristics could have biased our study. Observational design and the use of health services encounter data rather than clinical data, not weighing the statin vs. no statins groups, and the lack of biomarker measurements represents other limitations of the study.

In future studies, it will be relevant to measure markers of inflammation such as proinflammatory and anti-inflammatory cytokines, CRP, chemokines, neopterin measures of oxidative stress, markers of endothelial function, micro-RNA involved in proinflammatory and anti-inflammatory responses, physiological measurements of endothelial function, glucocorticosteroid measurement to consider effects of stress, acute and chronic, especially on TDO and immune function, tryptophan, kynurenine and molecules of the kynurenine pathway, precursors of dopamine affected by inflammation (phenylalanine, tyrosine, and their ratio), liver function tests (to monitor and account for statin toxicity), and of course, lipid profiles, especially total cholesterol, LDL cholesterol, and triglycerides. For large studies nested in electronic medical records, or for long-term monitoring, perhaps the most accessible and relevant clinical parameters are routine hematological laboratory results implicated in inflammation, such as WBC, neutrophil count, neutrophil/lymphocyte ratio, thrombocyte/lymphocyte ratio. The neutrophil:lymphocyte ratio (NLR) has been reported to be elevated in acute relapse inschizophrenia [168] and bipolar disorder [169],in particular in functionally relevant cognitive dysfunction and functional deterioration, as well as in increased suicide risk in euthymic bipolar individuals [170]. In tight experimental conditions, pretreatment with statins has been shown to prevent elevated NLR [171] after experimental stroke in rodents.

Future studies could be classified into two broad categories - studies of patients who have both metabolic/ cardiovascular indications and psychiatric indications and, probably at a later stage, studies on individuals who have only psychiatric indications, with incomplete responses to psychotropic medication. The outcome of the interventions could be psychiatric admissions (as done in this article), psychiatric instability (perhaps as a composite measure, including parameters related to stability-instability of mental illness, such as admissions, emergency room visits, need to increase medication dosages or replace medications, notable changes in the level of functioning [academic, occupational, activities of daily living, maintaining relationships], trouble with the law, loss of housing, remission, relapse, or recurrence of substance abuse, and nonadherence with psychiatric medications and follow-up, and with larger samples - suicide attempts, episodes of violence, or, with even larger samples - mandating multicenter collaboration - death by suicide). Either by design, or by analysis (adjustment, stratification, postrandomization weighing), the intervention and control groups (statin vs. no statin randomization, and lipophilic vs. hydrophilic statin) would have to take into account demographic factors (age and gender), metabolic/cardiovascular indications of statins, substance use, psychiatric diagnosis (schizophrenia or bipolar I), its severity and treatment, and history of immune-mediated conditions (autoimmune, allergic, severe or persistent infections, and TBI). In particular, baseline markers of immune activation could represent criteria of inclusion (i.e., in line with the idea that inflammation must be present to be valuable to modulate it) or stratification. Machine learning paradigms could be employed for both weighing factors that may contribute to the outcome, analyzing heterogeneity of effects (i.e., what combination of demographic, clinical, and pharmacological factors predict the strongest effect size, or in contrast little or no benefit, or side effects of statin treatment). In essence, it will be important to know not only if statins have benefits, but also which categories of statins, which dosages, and what durations manifest the greatest psychiatric benefits, and what are the broad characteristics of patients most likely to benefit from statin add-on treatment.

#### 4.4.8 Implications and future plans

In sum, this study identified that while both subgroups of statins are associated with lower RR of psychiatric hospitalization and ED visits, hydrophilic statins were advantageous in reducing the risk of psychiatric hospitalizations compared to lipophilic studies. The difference between hydrophilic and lipophilic statins was relatively small, and yet, given the proportion of individuals who are taking statins, this small effect may result in public health implications given the large number of individuals struggling with mental health issues, the increased metabolic and cardiovascular morbidity, and mortality in patients with severe mental illness.

Given the high proportion of Veterans with the history of mental illness who are being treated with a statin, the augmentation of psychotropic treatment in schizophrenia and BD with an individualized statin may have broad positive consequences. Understanding not only if statins help therapeutic controls but also what are the demographic, clinical, and pharmacologic characteristics of subgroups of patients who would benefit the most, and by which specific statin regimen (considering the lipophilia, potency, dose, and individual pharmacokinetic properties) is necessary. These efforts may lead to improved therapeutic control by repurposing statins as an add-on intervention in individuals in mental health treatment.

## 5 Conclusion

As pharmacological agents that engage multiple molecular targets implicated in onset, severity, and poor therapeutic control in schizophrenia and BD, personalized statin augmentation may become a salient, efficient, and nonstigmatizing intervention for reducing ER visits and hospitalization, achieving and sustaining remission, and maximizing functional recovery.

## Figures and Tables

**Table 1.** Description of individuals with schizophrenia on antipsychotic medication.

Schizophrenia with antipsychotic	Whole		Lipophilic		Hydrophilic		None	
	N=185449	SD or %	N=11293	SD or %	N=4759	SD or %	N=169397	SD or %
Mean age SD	58.67	9.99	58.49	9.5	58.18	9.38	58.7	10.03
Male	169985	91.66%	10480	92.80%	4388	92.20%	155117	91.57%
Race								
White	111513	60.13%	7007	62.05%	2893	60.79%	101613	59.99%
Non-White	65446	35.29%	3761	33.30%	1642	34.50%	60043	35.45%
Missing	8490	4.58%	525	4.65%	224	4.71%	7741	4.57%
Ever married	124317	67.04%	7514	66.54%	3279	68.90%	113524	67.02%
with service percentage >=50%	102312	55.17%	6149	54.45%	2661	55.92%	93502	55.20%
Fiscal year								
FY11	24502	13.21%	997	8.83%	893	18.76%	22612	13.35%
FY12	49032	26.44%	1660	14.70%	2250	47.28%	45122	26.64%
FY13	47452	25.59%	3903	34.56%	843	17.71%	42706	25.21%
FY14	43940	23.69%	3371	29.85%	547	11.49%	40022	23.63%
FY15	20523	11.07%	1362	12.06%	226	4.75%	18935	11.18%
Psychiatric condition								
major depression	45121	24.33%	2613	23.14%	1081	22.71%	41427	24.46%
PTSD	33771	18.21%	1913	16.94%	838	17.61%	31020	18.31%
Alcohol use disorder	36316	19.58%	1792	15.87%	731	15.36%	33793	19.95%
Other substance use disorder	34198	18.44%	1671	14.80%	660	13.87%	31867	18.81%
Psychiatric medication use								
Prescribed ATP	185449	100%	11293	100%	4759	100%	169397	100%
Antipsychotics coverage >=80%	104434	56.31%	6431	56.95%	2857	60.03%	95146	56.17%
Prescribed clozapine	7473	4.03%	411	3.64%	159	3.34%	6903	4.08%
Prescribed mood stabilizer medication	75709	40.82%	4204	37.23%	1829	38.43%	69676	41.13%
Mood stabilizer medication coverage >=80%	37797	20.38%	2155	19.08%	942	19.79%	34700	20.48%
Prescribed antidepressant	118310	63.80%	7094	62.82%	3044	63.96%	108172	63.86%
Prescribed anti-anxiety medication	77130	41.59%	4309	38.16%	1873	39.36%	70948	41.88%
Prescribed substance use disorder medication	8957	4.83%	430	3.81%	175	3.68%	8352	4.93%
Service use								
Hospitalized for somatic reason	34769	18.75%	1557	13.79%	590	12.40%	32622	19.26%
Emergency room use	65965	35.57%	3241	28.70%	1263	26.54%	61461	36.28%
PRRC visits	15.41	2.38	14.59	2.26	16.5	2.59	15.43	2.38
MHICM visits	13	4.22	12.2	3.78	12.45	3.72	13.07	4.26
Other mental health outpatient visits	12.8	7.63	12.6	7.07	13.48	7.12	12.79	7.68
Substance use visits	8.69	1.39	7.07	1.06	7.68	1.08	8.81	1.43
Somatic outpatient visits	18.36	18.22	16.55	15.79	17.15	16.43	18.5	18.44
Somatic outpatient visits during 3M post	11.57	11.56	10.06	9.74	9.45	9.56	11.7	11.74
Carlson Comorbidity Index			4524	40.06%	1839	38.64%	64872	38.30%
0	71235	38.41%	4007	35.48%	1700	35.72%	57702	34.06%
1	63409	34.19%	2762	24.46%	1220	25.64%	46823	27.64%
2+	50805	27.40%	9.5	58.49	9.38	58.18	10.03	58.7

FY: Fiscal year in which incident medication episode began; PTSD: Post- Traumatic Stress Disorder; ATP: Prescribed Anti-Psychotic; PPRC: Count of Psychosocial Rehabilitation and Recovery Center visits; MHICM: Count of Mental Health Intensive Case Management visits.

**Table 2.** Description of individuals with BD on antipsychotic medication.

Bipolar with antipsychotic	Whole		Lipophilic		Hydrophilic		None	
	N=211412	SD or %	N=13292	SD or %	N=5326	SD or %	N=192794	SD or %
Mean age SD	54.77	11.29	55.93	10.37	56.11	10.12	54.65	11.37
Male	174106	82.35%	11424	85.95%	4519	84.85%	158163	82.04%
Race								
White	169511	80.18%	10919	82.15%	4397	82.56%	154195	79.98%
Non-White	34040	16.1%	1843	13.87%	737	13.84%	31460	16.32%
Missing	7861	3.72%	530	3.99%	192	3.60%	7139	3.70%
Ever married	178906	84.62%	11373	85.56%	4644	87.19%	162889	84.49%
with service percentage >=50%	93123	44.05%	5654	42.54%	2226	41.79%	85243	44.21%
Fiscal year								
FY11	25504	12.06%	1151	8.66%	1041	19.55%	23312	12.09%
FY12	53920	25.5%	1864	14.02%	2374	44.57%	49682	25.77%
FY13	54168	25.62%	4729	35.58%	1018	19.11%	48421	25.12%
FY14	52605	24.88%	3931	29.57%	637	11.96%	48037	24.92%
FY15	25215	11.93%	1617	12.17%	256	4.81%	23342	12.11%
Psychiatric condition								
major depression	81864	38.72%	4732	35.60%	1846	34.66%	75286	39.05%
PTSD	79552	37.63%	4483	33.73%	1827	34.30%	73242	37.99%
Alcohol use disorder	56951	26.94%	3016	22.69%	1118	20.99%	52817	27.40%
Other substance use disorder	50489	23.88%	2530	19.03%	980	18.40%	46979	24.37%
Psychiatric medication use								
Prescribed ATP	211412	100%	13292	100%	5326	100%	192794	100%
Antipsychotics coverage >=80%	90831	42.96%	6001	45.15%	2491	46.77%	82339	42.71%
Prescribed clozapine	361	0.17%	16	0.12%	8	0.15%	337	0.17%
Prescribed mood stabilizer medication	137213	64.9%	8426	63.39%	3435	64.49%	125352	65.02%
Mood stabilizer medication coverage >=80%	65707	31.08%	4239	31.89%	1725	32.39%	59743	30.99%
Prescribed antidepressant	159677	75.53%	9834	73.98%	3970	74.54%	145873	75.66%
Prescribed anti-anxiety medication	111319	52.66%	6459	48.59%	2718	51.03%	102142	52.98%
Prescribed substance use disorder medication	22158	10.48%	1129	8.49%	440	8.26%	20589	10.68%
Service use								
Hospitalized for somatic reason	36434	17.23%	1748	13.15%	628	11.79%	34058	17.67%
Emergency room use	76432	36.15%	3905	29.38%	1474	27.68%	71053	36.85%
PRRC visits	8.93	0.92	8.42	0.88	9.1	0.94	8.96	0.92
MHICM visits	6.36	0.96	5.82	0.83	5.51	0.8	6.42	0.97
Other mental health outpatient visits	14.37	8.76	13.18	7.7	12.18	7.23	14.5	8.88
Substance use visits	11.11	2.37	10	1.89	9.09	1.63	11.23	2.43
Somatic outpatient visits	18	18.96	16.47	16.27	16.33	16.98	18.12	19.2
Somatic outpatient visits during 3M post	11.88	12.15	10.29	9.9	9.91	9.89	12.01	12.37
Carlson Comorbidity Index								
0	96283	45.54%	6139	46.19%	2337	43.88%	87807	45.54%
1	67107	31.74%	4316	32.47%	1815	34.08%	60976	31.63%
2+	48022	22.71%	2837	21.34%	1174	22.04%	44011	22.83%

FY: Fiscal year in which incident medication episode began; PTSD: Post- Traumatic Stress Disorder; ATP: Prescribed Anti-Psychotic; PPRC: Count of Psychosocial Rehabilitation and Recovery Center visits; MHICM: Count of Mental Health Intensive Case Management visits.

**Table 3.** Description of individuals with BD on mood-stabilizer medication.

Bipolar with mood stabilizer	Whole		Lipophilic		Hydrophilic		Other	
	N=286268	SD or %	N=18267	SD or %	N=7307	SD or %	N=260694	SD or %
Mean age SD	55.41	11.34	56.8	10.3	56.87	10.13	55.28	11.43
Male	238625	83.36%	15976	87.46%	6302	86.25%	216347	82.99%
Race								
White	233821	81.68%	15221	83.33%	6104	83.54%	212496	81.51%
Non-White	41238	14.41%	2308	12.63%	898	12.29%	38032	14.59%
Missing	11209	3.92%	738	4.04%	305	4.17%	10166	3.9%
Ever married	246998	86.28%	15971	87.43%	6502	88.98%	224525	86.13%
with service percentage >=50%	126007	44.02%	7867	43.07%	2990	40.92%	115150	44.17%
Fiscal year								
FY11	32946	11.51%	1454	7.96%	1418	19.41%	30074	11.54%
FY12	71876	25.11%	2478	13.57%	3346	45.79%	66052	25.34%
FY13	73489	25.67%	6626	36.27%	1269	17.37%	65594	25.16%
FY14	73066	25.52%	5457	29.87%	896	12.26%	66713	25.59%
FY15	34891	12.19%	2252	12.33%	378	5.17%	32261	12.38%
Psychiatric condition								
major depression	109158	38.13%	6404	35.06%	2503	34.25%	100251	38.46%
PTSD	100897	35.25%	5878	32.18%	2314	31.67%	92705	35.56%
Alcohol use disorder	69117	24.14%	3678	20.13%	1366	18.69%	64073	24.58%
Other substance use disorder	59663	20.84%	2964	16.23%	1072	14.67%	55627	21.34%
Psychiatric medication use								
Prescribed ATP	137213	47.93%	8426	46.13%	3435	47.01%	125352	48.08%
Antipsychotics coverage >=80%	60215	21.03%	3909	21.4%	1600	21.9%	54706	20.98%
Prescribed clozapine	277	0.1%	13	0.07%	4	0.05%	260	0.1%
Prescribed mood stabilizer medication	286268	100%	18267	100%	7307	100%	260694	100%
Mood stabilizer medication coverage >=80%	129612	45.28%	8582	46.98%	3489	47.75%	117541	45.09%
Prescribed antidepressant	211314	73.82%	13191	72.21%	5299	72.52%	192824	73.97%
Prescribed anti-anxiety medication	142628	49.82%	8294	45.4%	3471	47.5%	130863	50.2%
Prescribed substance use disorder medication	26805	9.36%	1309	7.17%	519	7.1%	24977	9.58%
Service use								
Hospitalized for somatic reason	49987	17.46%	2470	13.52%	907	12.41%	46610	17.88%
Emergency room use	101884	35.59%	5441	29.79%	2050	28.06%	94393	36.21%
PRRC visits	7.7	0.71	7.45	0.68	7.27	0.61	7.73	0.71
MHICM visits	5.17	0.64	4.61	0.54	4.58	0.55	5.22	0.65
Other mental health outpatient visits	13.71	7.96	12.2	6.83	11.36	6.38	13.87	8.08
Substance use visits	10.65	2.16	9.67	1.65	8.92	1.4	10.76	2.22
Somatic outpatient visits	18.45	19.88	16.63	17.21	16.95	17.85	18.59	20.12
Somatic outpatient visits during 3M post	12.2	12.64	10.61	10.52	10.42	10.51	12.33	12.85
Carlson Comorbidity Index								
0	125230	43.75%	7964	43.6%	3035	41.54%	114231	43.82%
1	90077	31.47%	5947	32.56%	2461	33.68%	81669	31.33%
2+	70961	24.79%	4356	23.85%	1811	24.78%	64794	24.85%

FY: Fiscal year in which incident medication episode began; PTSD: Post- Traumatic Stress Disorder; ATP: Prescribed Anti-Psychotic; PPRC: Count of Psychosocial Rehabilitation and Recovery Center visits; MHICM: Count of Mental Health Intensive Case Management visits.

**Table 4.** Inpatient psychiatric hospitalization in individuals with schizophrenia and bipolar disorder; comparison between lipophilic, hydrophilic and no statin groups.

Label		Relative Risk	Mean		L’Beta Estimate	Standard Error	Alpha	L’Beta		Chi-Square	P
			Confidence Limit					Confidence Limit			
**Lipophilic vs Hydrophilic**	Schizophrenia with anti-psychotic	1.11	1.0007	1.23	0.1	0.05	0.05	0.0007	0.21	3.89	0.048
	BD with antipsychotic	1.22	1.09	1.36	0.2	0.06	0.05	0.08	0.31	11.42	<.001
	BD with mood-stabilizer	1.18	1.06	1.32	0.17	0.05	0.05	0.06	0.27	9.37	0.002
**Lipophilic vs None**	Schizophrenia with antipsychotic	0.81	0.77	0.86	−0.21	0.03	0.05	−0.26	−0.16	59.84	<.001
	BD with antipsychotic	0.85	0.80	0.89	−0.17	0.03	0.05	−0.22	−0.11	36.88	<.001
	BD with mood-stabilizer	0.83	0.79	0.88	−0.18	0.03	0.05	−0.23	−0.13	49.31	<.001
**Hydrophilic vs None**	Schizophrenia with antipsychotic	0.73	0.67	0.80	−0.31	0.05	0.05	−0.40	−0.22	47.16	<.001
	BD with antipsychotic	0.70	0.63	0.77	−0.36	0.05	0.05	−0.46	−0.26	50.07	<.001
	BD with mood-stabilizer	0.70	0.64	0.77	−0.35	0.05	0.05	−0.44	−0.26	53.89	<.001

BD: Bipolar Disorder

**Table 5.** Psychiatric emergency department visit in individuals with schizophrenia and bipolar disorder; comparison between lipophilic, hydrophilic and no statin groups.

Label		Relative Risk	Mean		L’Beta Estimate	Standard Error	Alpha	L’Beta		Chi-Square	P
			Confidence Limit					Confidence Limit			
**Lipophilic vs Hydrophilic**	Schizophrenia with antipsychotic	1.07	0.83	1.36	0.06	0.13	0.05	−0.18	0.31	0.27	0.61
	BD with antipsychotic	1.26	0.97	1.63	0.23	0.13	0.05	−0.03	0.49	3.11	0.08
	BD with mood-stabilizer	1.16	0.92	1.46	0.15	0.12	0.05	−0.08	0.38	1.63	0.2
**Lipophilic vs None**	Schizophrenia with antipsychotic	0.76	0.67	0.87	−0.27	0.07	0.05	−0.41	−0.14	16.44	<.001
	BD with antipsychotic	0.83	0.74	0.94	−0.18	0.06	0.05	−0.31	−0.06	8.44	0.004
	BD with mood-stabilizer	0.79	0.71	0.89	−0.23	0.06	0.05	−0.35	−0.12	16.09	<.001
**Hydrophilic vs None**	Schizophrenia with antipsychotic	0.71	0.58	0.88	−0.34	0.11	0.05	−0.55	−0.13	10.03	0.002
	BD with antipsychotic	0.66	0.53	0.83	−0.41	0.12	0.05	−0.64	−0.19	12.93	<0.001
	BD with mood-stabilizer	0.68	0.56	0.83	−0.38	0.1	0.05	−0.58	−0.18	14.09	<0.001

BD: Bipolar Disorder

## Data Availability

The data are not available for direct sharing considering that the data do not belong to the authors, but to the Veterans Health Administration, and there is no provision approved by Institutional Review Board (IRB). Interested individuals should contact the authors via email of possible collaborations.
